# Supplementary material for: A stress-dependent TDP-43 SUMOylation program preserves neuronal function
Source: Mol Neurodegener. 2025 Mar 28;20:38. doi: 10.1186/s13024-025-00826-z (PMC11951803; doi:10.1186/s13024-025-00826-z)
Supplement: Supplementary file 5 — Supplementary Material 5. Fig. S1: Characterizing stress responsive TDP-43 SUMOylation. (A) Representative immunofluorescent microscopy images of TDP-43 and G3BP1 and western blot from SUMOylation assays testing whether various stressors that cause TDP-43 aggregation induce TDP-43 SUMOylation: NaAsO2 (250 μM), D-Sorbitol (400 mM), NaCl (300 mM), Heat Shock (42 °C) in HEK293T (Immunofluorescence) and HEK293T HA-SUMO2 stable cells (biochemistry). One-Way ANOVA with Fisher’s LSD test. Data presented as mean ± SEM. *p <0.05, **p <0.005, ****p <0.0001. (B) Representative western blot from SUMOylation assays testing the selectivity of SUMO paralogs to SUMOylate TDP-43 in response to 1 hour sodium arsenite (250 μM) in HEK293T cells with transient expression of SUMO paralogs. (C) Representative GFP-Trap SUMOylation assay depicting increased SUMOylation with ALS-causing Q331K mutation in response to 250 µM sodium arsenite stress in HEK293T cells with transient expression of HA-SUMO2. Unpaired T-test. Data presented as mean ± SEM relative to stressed TDP-43-GFP (WT) condition, *p <0.05. (D) Representative images of SUMO2/3 and TDP-43 in the nucleus of mouse cortical neurons (7 DIV) in unstressed and 1 hour sodium arsenite (250 μM) stressed conditions. (E) Representative control imaged from Proximity Ligation Assay (PLA) in mouse primary cortical neurons. Scale bar = 20 μm. (N = 4). (F) Representative western blot and quantification from GFP-Trap SUMOylation assays testing the kinetics of TDP-43 SUMOylation in response to sodium arsenite stress (250 μM) and its relationship to insoluble phosphorylated TDP-43 in HEK293T HA-SUMO2 stable cells. (N = 3). Data presented as mean ± SEM relative to 3-hour stress condition. Linear regression analysis of Relative SUMOylation against time in response to stress. (G) Representative dose-response assay to detect TDP-43 SUMOylation after 1 hour of sodium arsenite stress (1 = 250 μM, 1/2 = 125 μM, 1/4 = 62.5 μM, etc.) in HEK293T HA-SUMO2 stab [file 13024_2025_826_MOESM5_ESM.zip › 13024_2025_826_MOESM5_ESM.pdf]

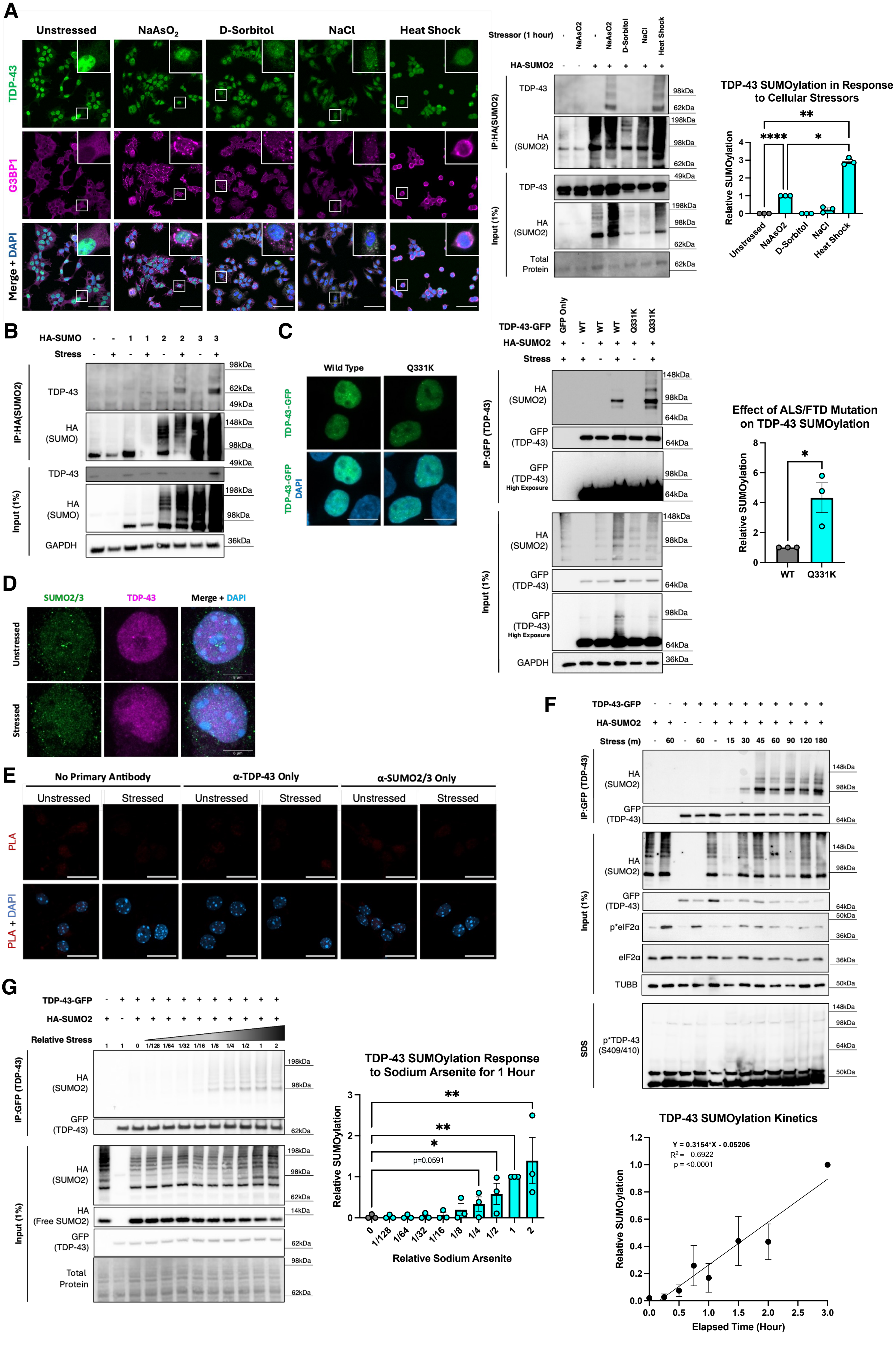

Fig. S1: Characterizing stress responsive TDP-43 SUMOylation.

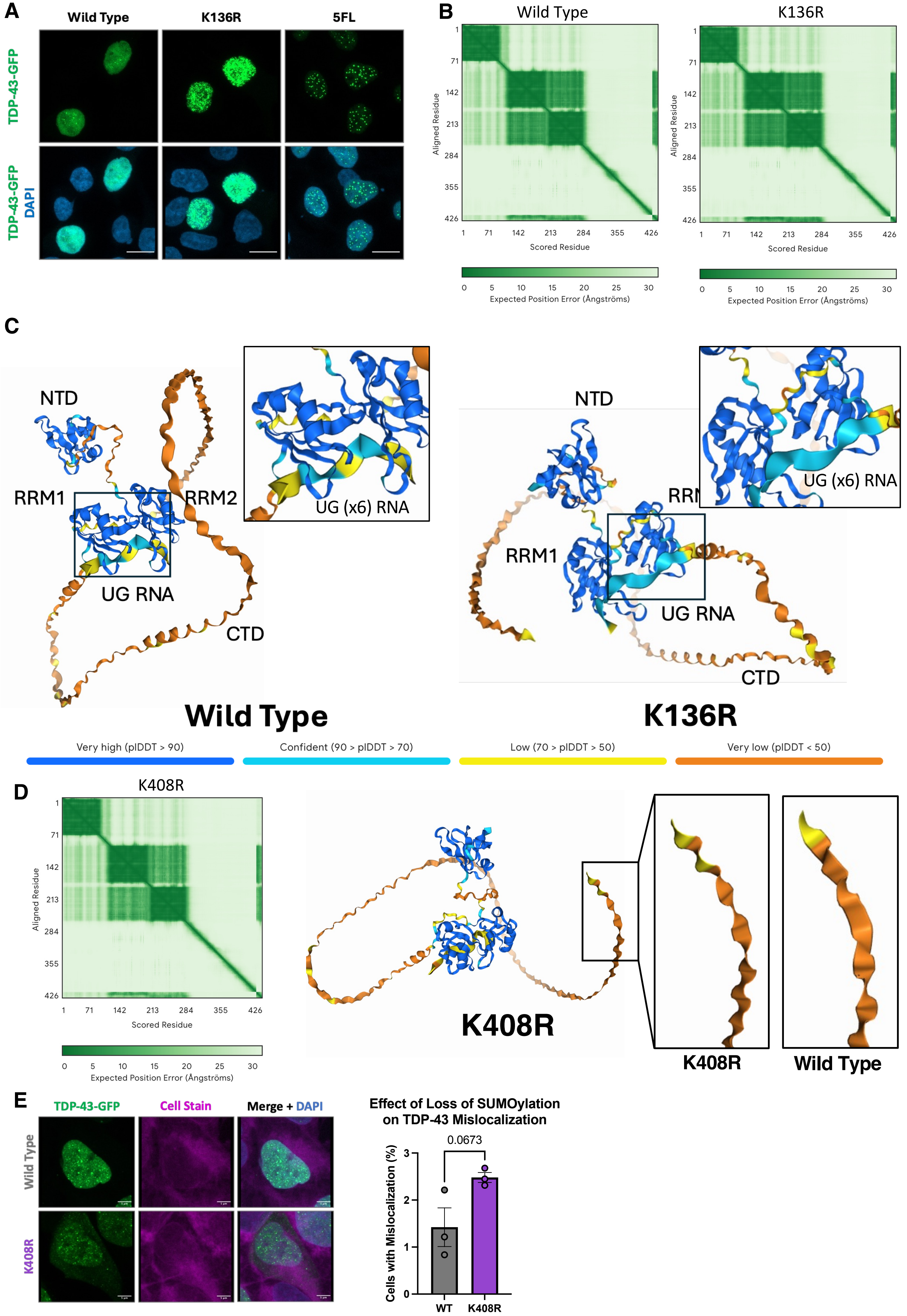

Fig. S2: Analysis of TDP-43 lysine to arginine mutation at K136 and K408.

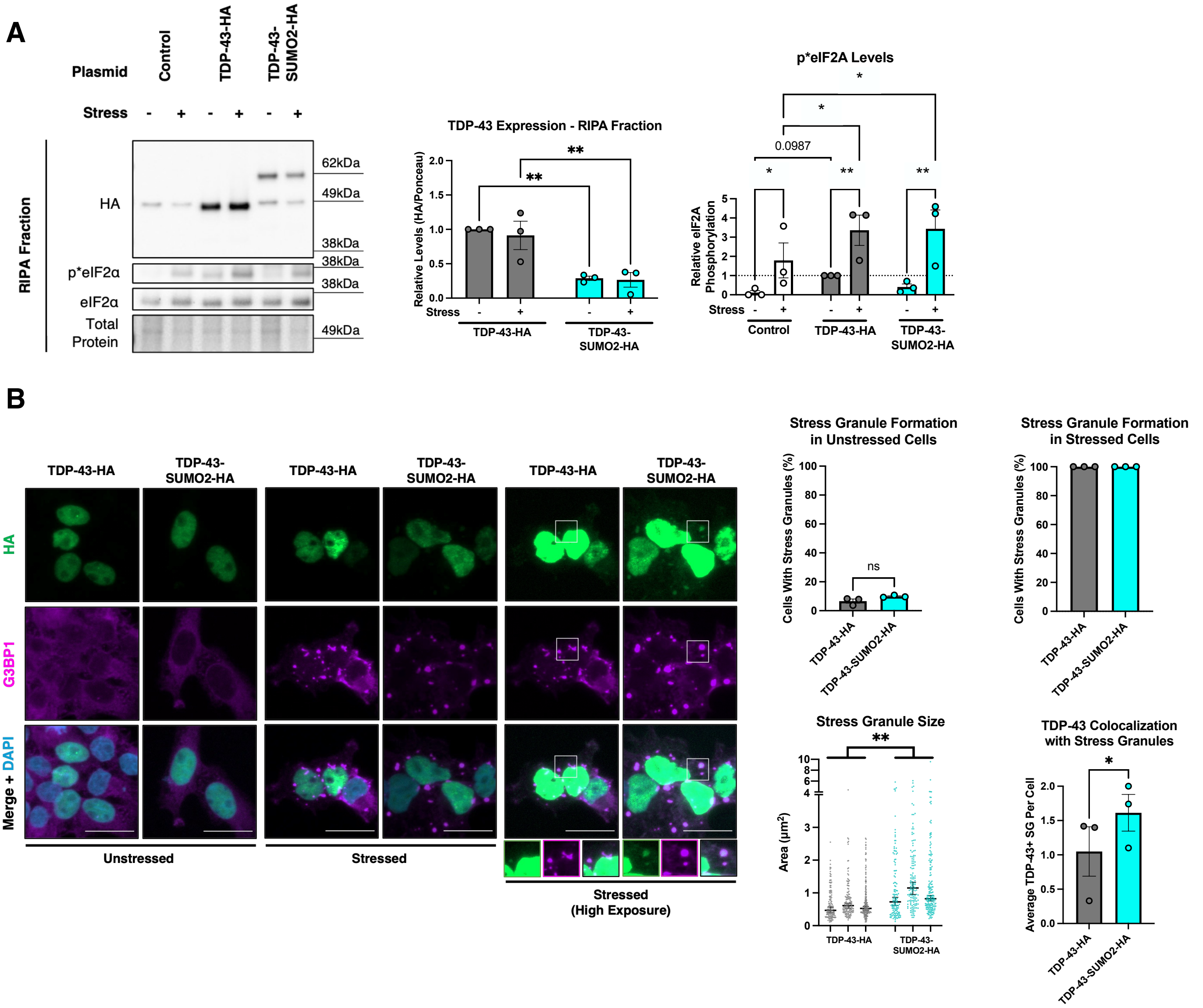

Fig. S3: Characterization of SUMO2 fusion to the C-terminus of TDP-43.

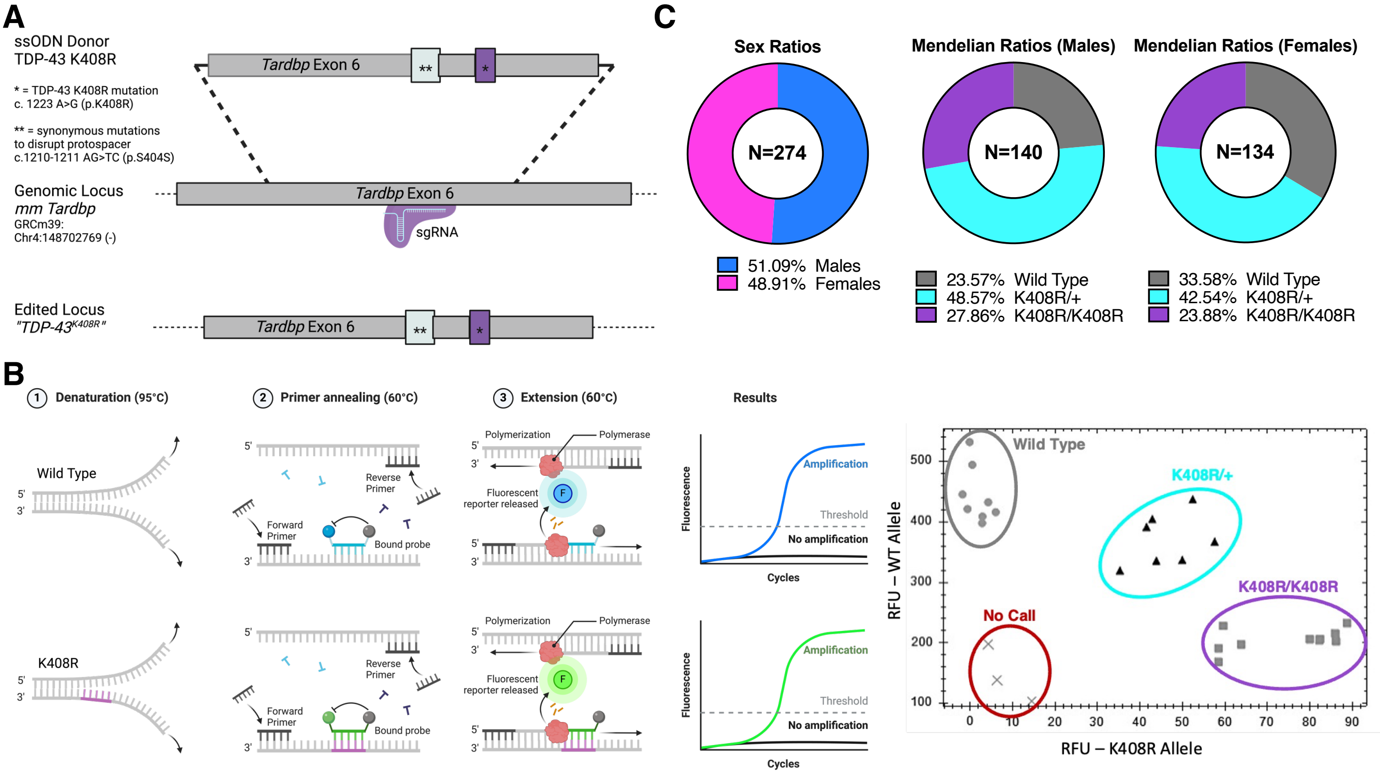

Fig. S4: Generation of the TDP-43^{K408R} mouse line.

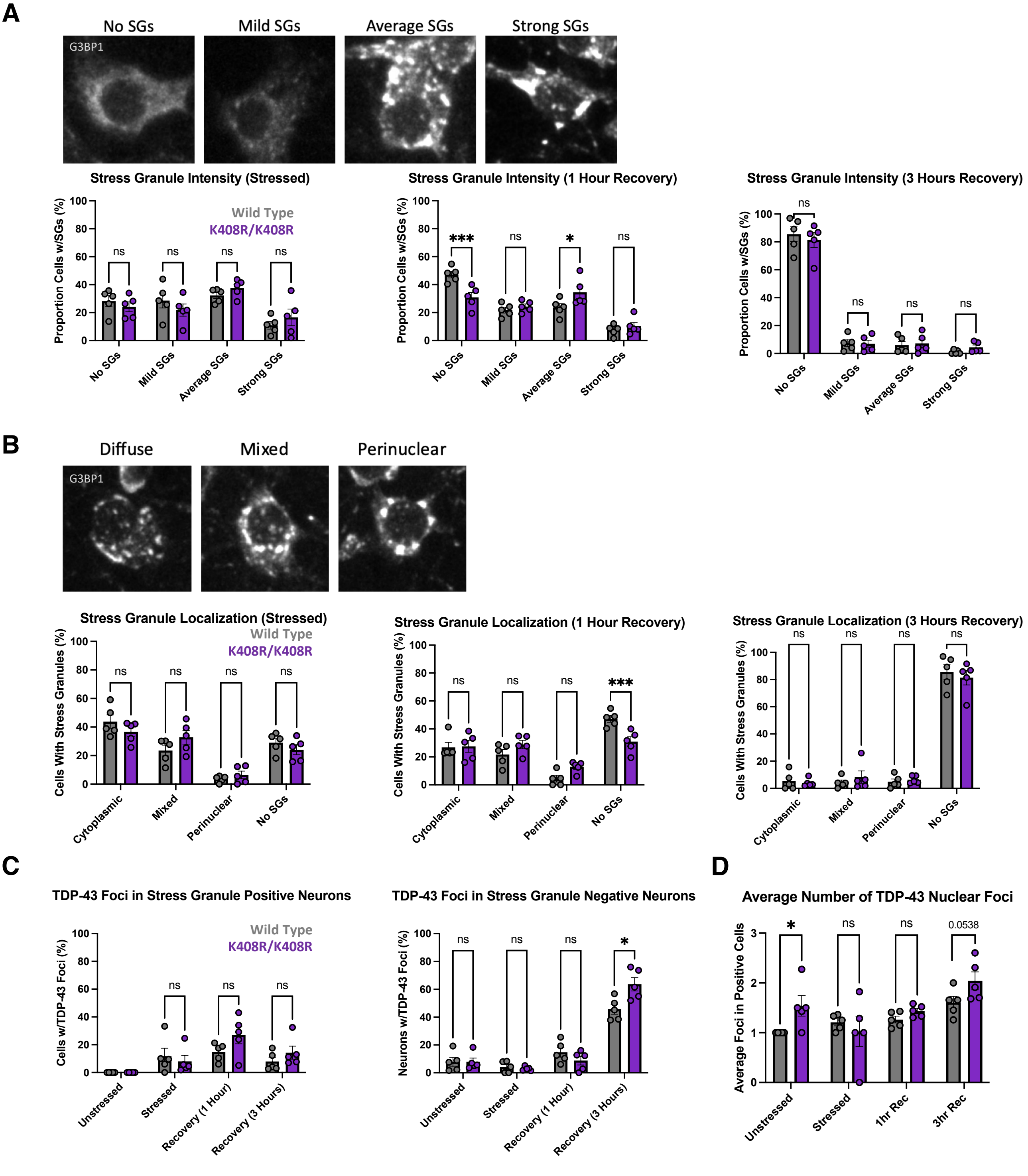

Fig. S5: Characterizing the stress response in TDP-43^{K408R} primary cortical neuron cultures.

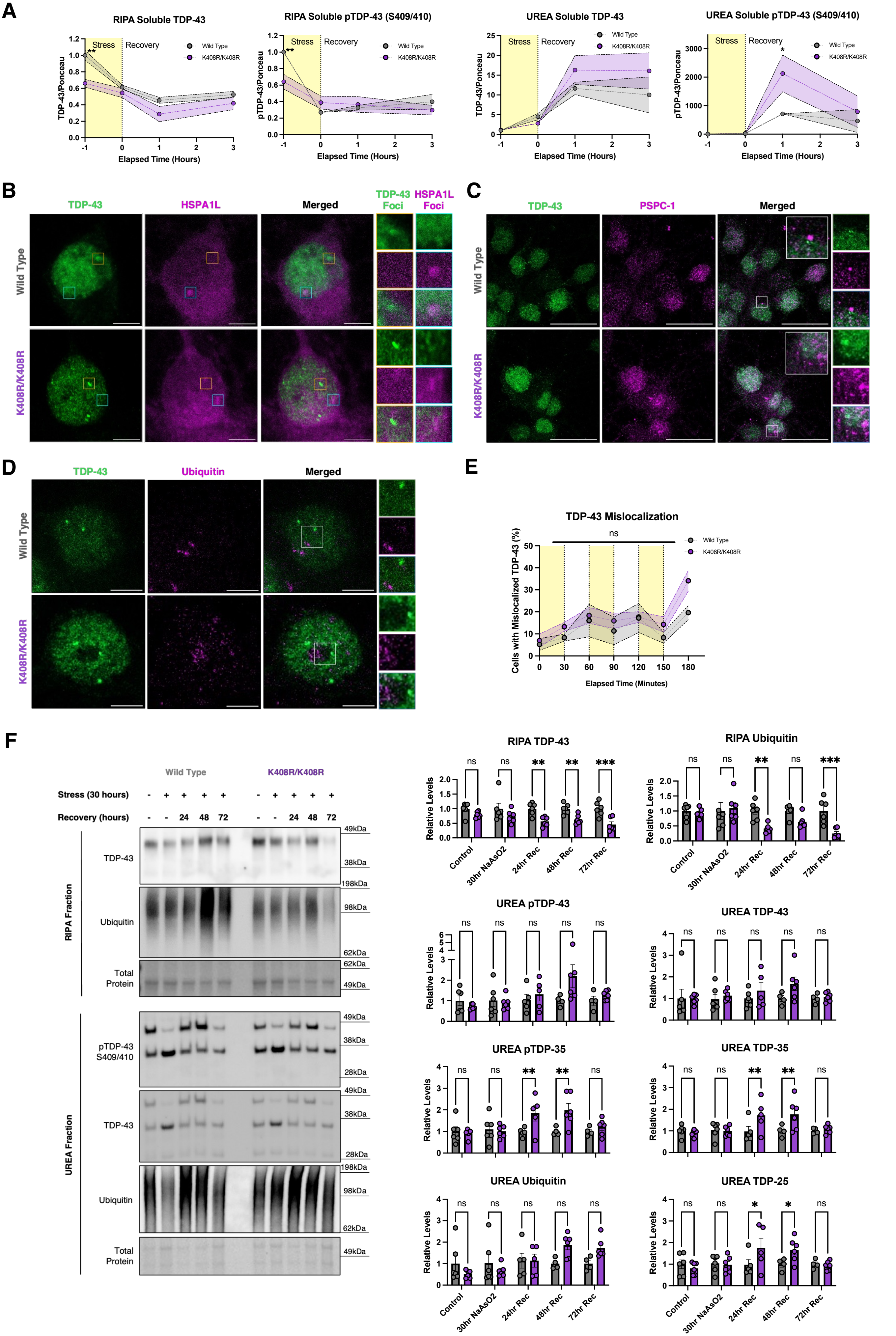

Fig. S6: Characterizing the effect of blocking TDP-43 SUMOylation in primary cortical neurons.

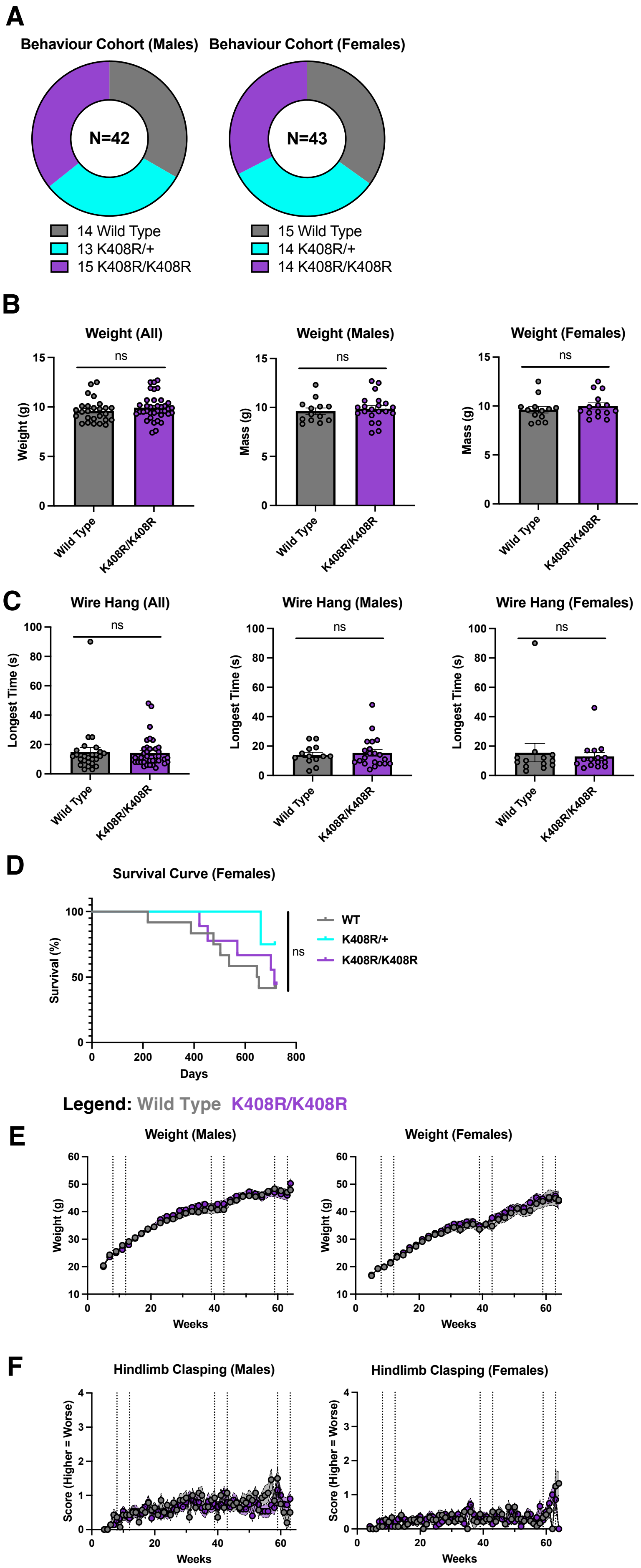

Fig. S7: General wellness of TDP-43^{K408R} mice during development and aging.

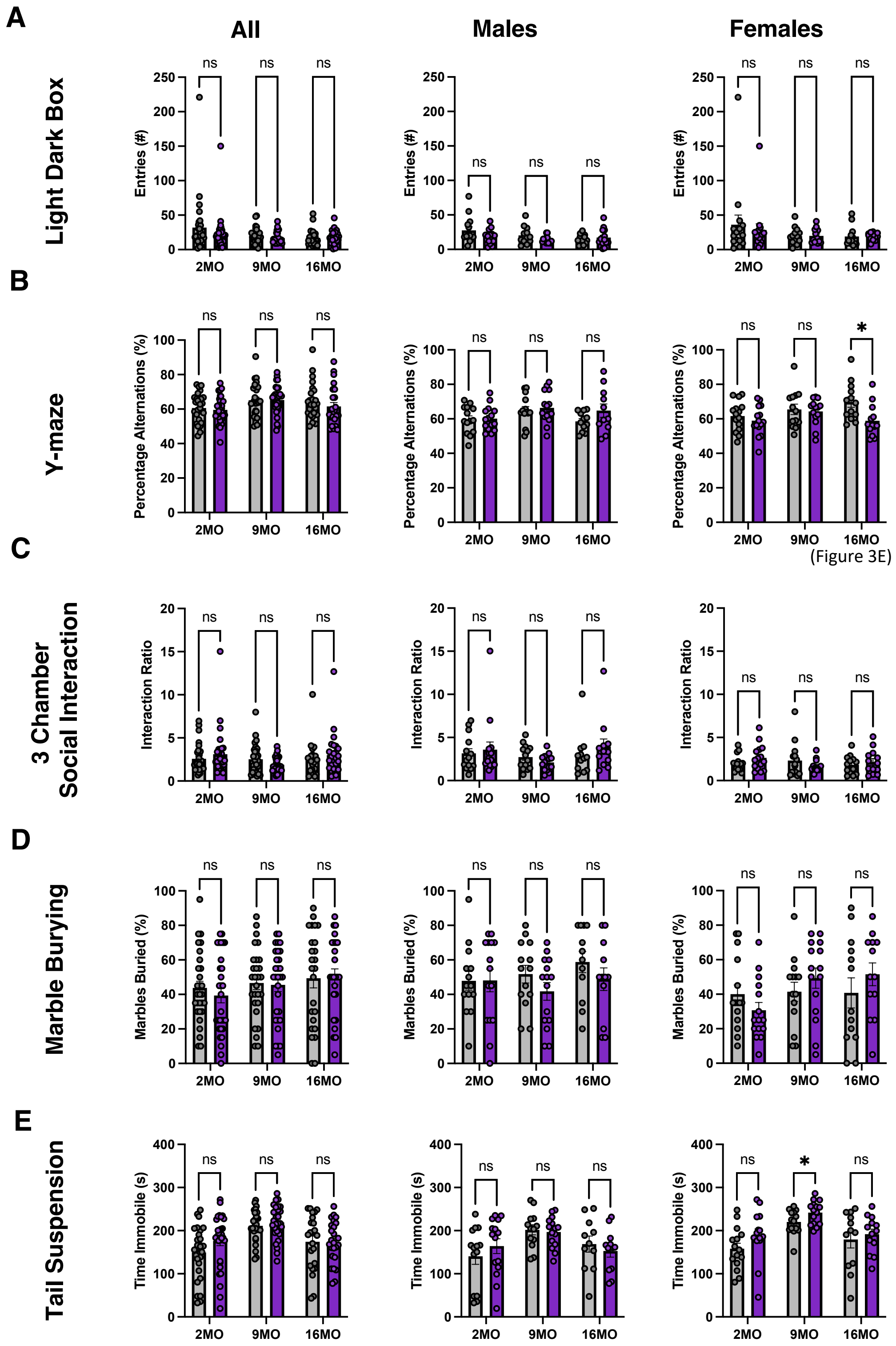

Fig. S8: Characterizing the cognitive and social behaviour of TDP-43^{K408R} mice.

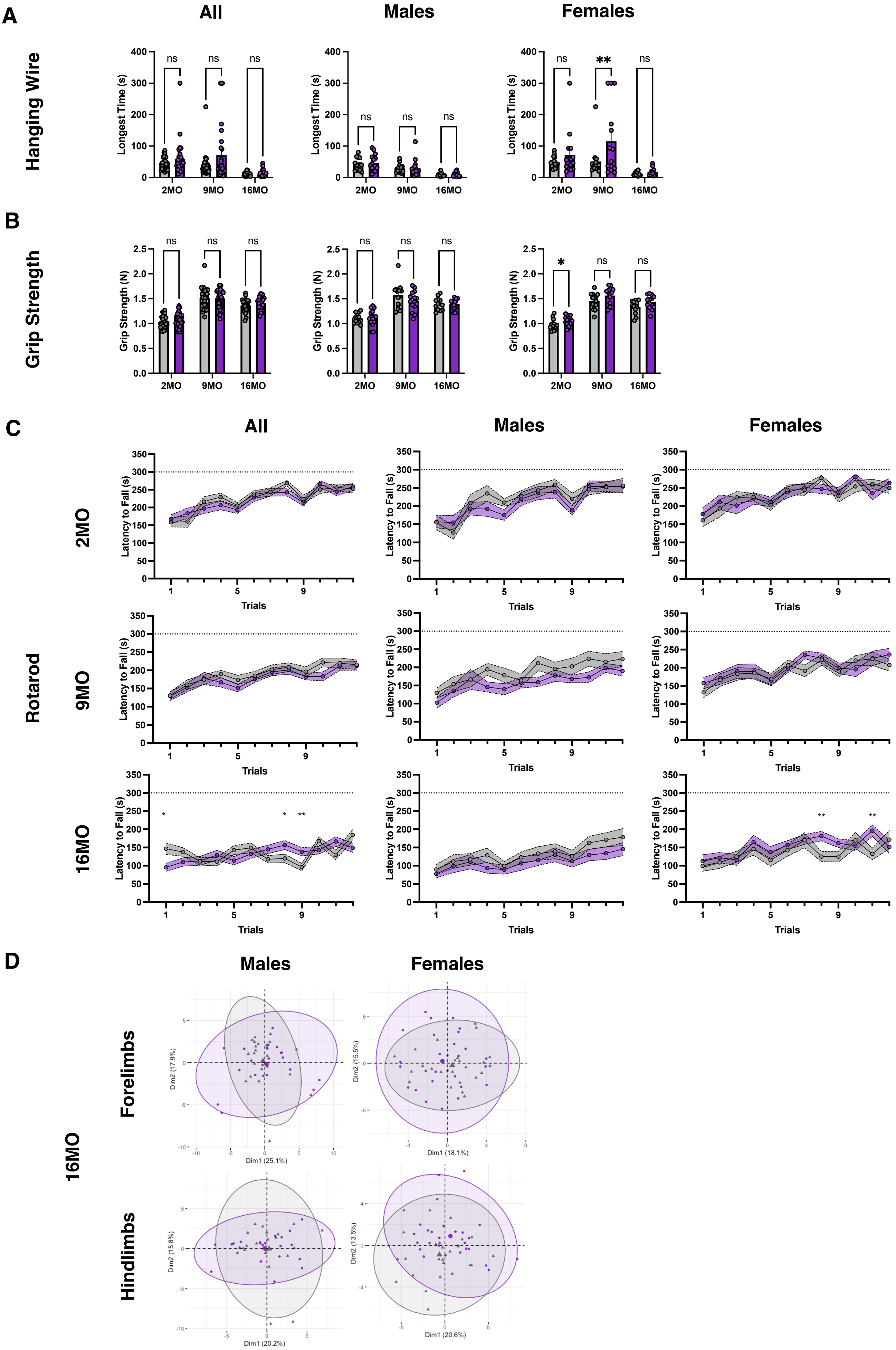

Fig. S9: Characterizing the motor behaviour of TDP-43^{K408R} mice.

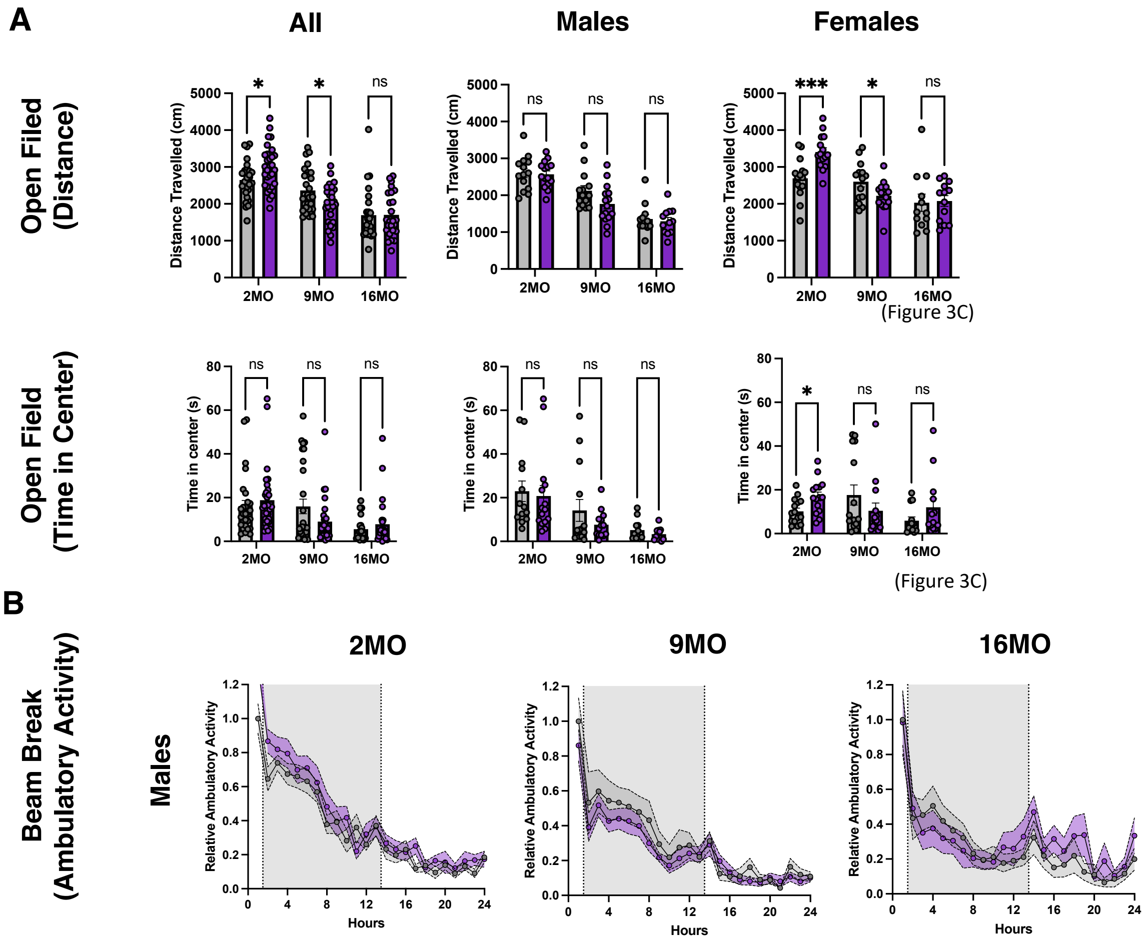

Fig. S10: Characterizing the activity of TDP-43^{K408R} mice.

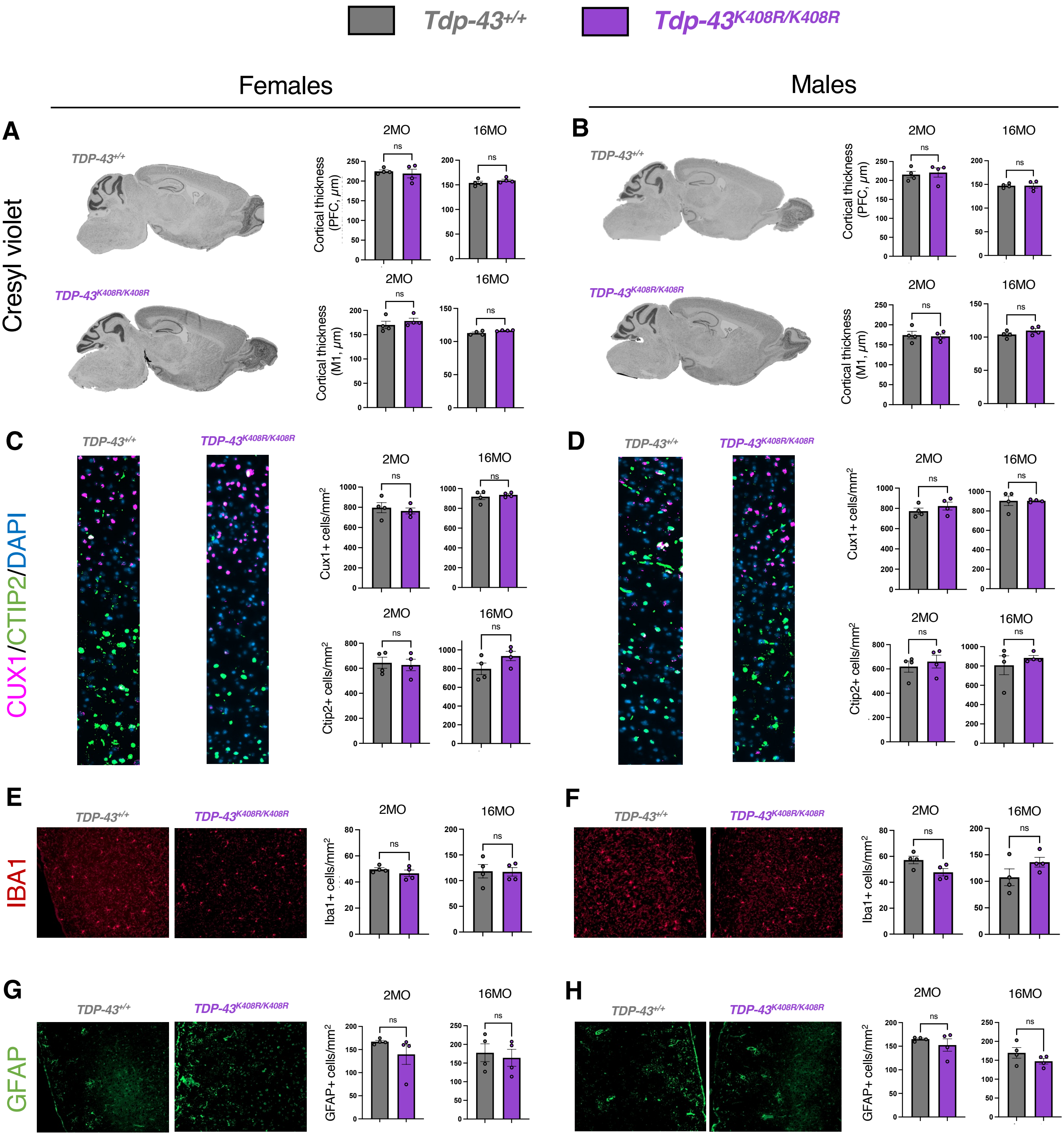

Fig. S11: Absence of overt cortical neurodegeneration or neuroinflammatory phenotypes in TDP-43$^{K408R/K408R}$ mice

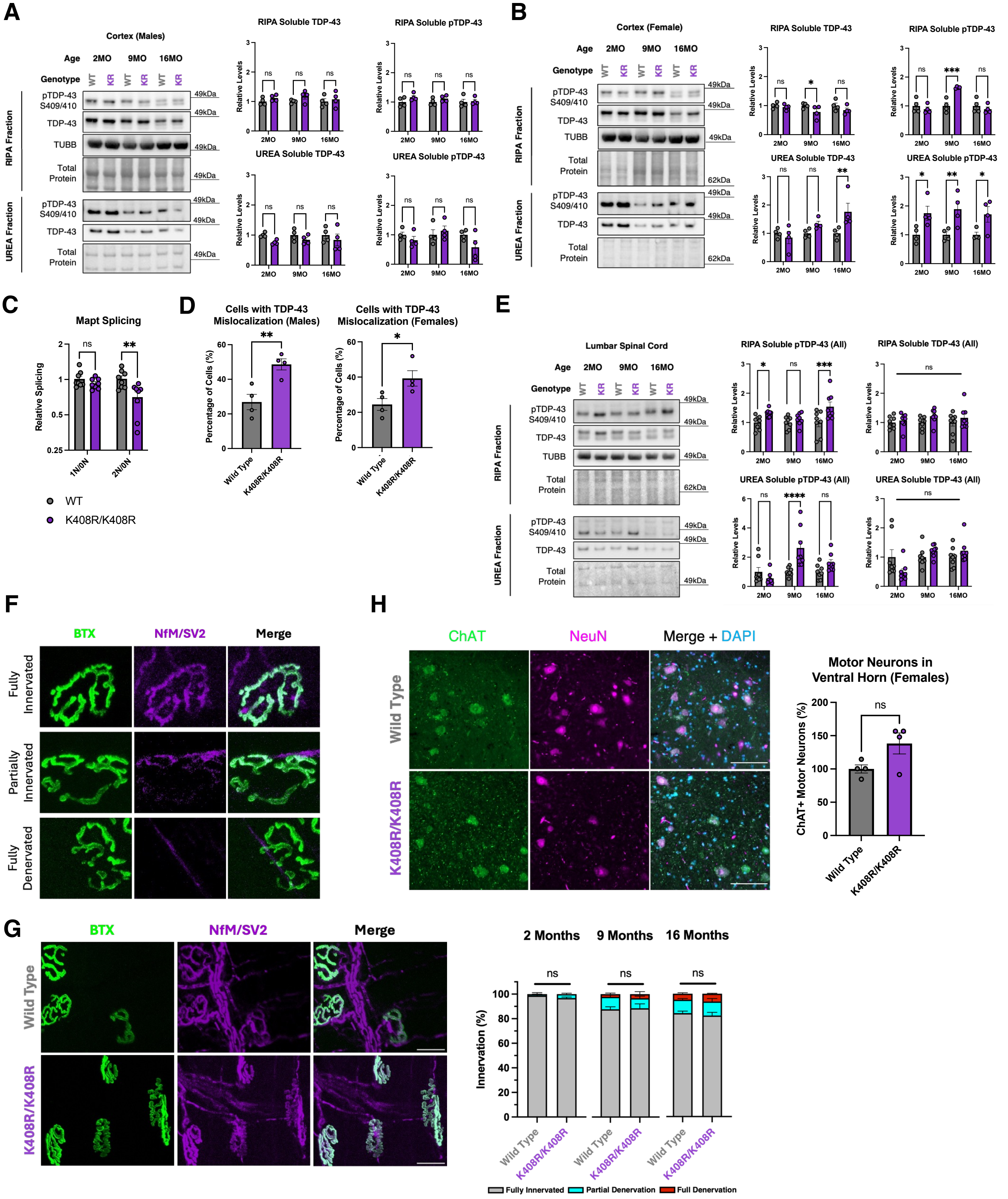

Fig. S12: Characterizing the molecular and histological effects of blocking TDP-43 SUMOylation at K408 *in vivo*.

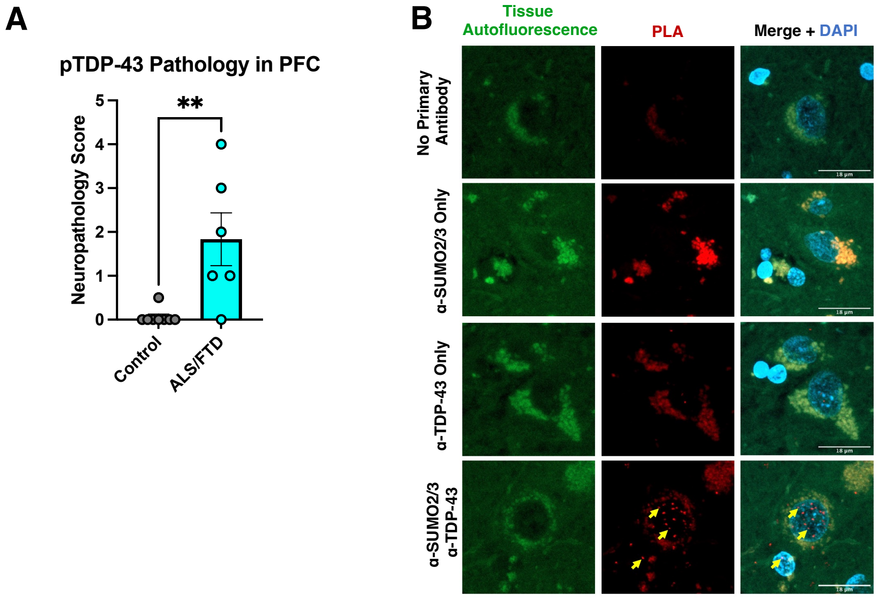

Fig. S13: Optimization of proximity ligation assay in human tissue.
