# Supplementary material for: A stress-dependent TDP-43 SUMOylation program preserves neuronal function
Source: Mol Neurodegener. 2025 Mar 28;20:38. doi: 10.1186/s13024-025-00826-z (PMC11951803; doi:10.1186/s13024-025-00826-z)
Supplement: Supplementary file 5 — Supplementary Material 5. Fig. S1: Characterizing stress responsive TDP-43 SUMOylation. (A) Representative immunofluorescent microscopy images of TDP-43 and G3BP1 and western blot from SUMOylation assays testing whether various stressors that cause TDP-43 aggregation induce TDP-43 SUMOylation: NaAsO2 (250 μM), D-Sorbitol (400 mM), NaCl (300 mM), Heat Shock (42 °C) in HEK293T (Immunofluorescence) and HEK293T HA-SUMO2 stable cells (biochemistry). One-Way ANOVA with Fisher’s LSD test. Data presented as mean ± SEM. *p <0.05, **p <0.005, ****p <0.0001. (B) Representative western blot from SUMOylation assays testing the selectivity of SUMO paralogs to SUMOylate TDP-43 in response to 1 hour sodium arsenite (250 μM) in HEK293T cells with transient expression of SUMO paralogs. (C) Representative GFP-Trap SUMOylation assay depicting increased SUMOylation with ALS-causing Q331K mutation in response to 250 µM sodium arsenite stress in HEK293T cells with transient expression of HA-SUMO2. Unpaired T-test. Data presented as mean ± SEM relative to stressed TDP-43-GFP (WT) condition, *p <0.05. (D) Representative images of SUMO2/3 and TDP-43 in the nucleus of mouse cortical neurons (7 DIV) in unstressed and 1 hour sodium arsenite (250 μM) stressed conditions. (E) Representative control imaged from Proximity Ligation Assay (PLA) in mouse primary cortical neurons. Scale bar = 20 μm. (N = 4). (F) Representative western blot and quantification from GFP-Trap SUMOylation assays testing the kinetics of TDP-43 SUMOylation in response to sodium arsenite stress (250 μM) and its relationship to insoluble phosphorylated TDP-43 in HEK293T HA-SUMO2 stable cells. (N = 3). Data presented as mean ± SEM relative to 3-hour stress condition. Linear regression analysis of Relative SUMOylation against time in response to stress. (G) Representative dose-response assay to detect TDP-43 SUMOylation after 1 hour of sodium arsenite stress (1 = 250 μM, 1/2 = 125 μM, 1/4 = 62.5 μM, etc.) in HEK293T HA-SUMO2 stab [file 13024_2025_826_MOESM5_ESM.zip › Supplemental Figure Captions.docx]

**Supplemental Figures**

**Fig. S1: Characterizing stress responsive TDP-43 SUMOylation. (A)** Representative immunofluorescent microscopy images of TDP-43 and G3BP1 and western blot from SUMOylation assays testing whether various stressors that cause TDP-43 aggregation induce TDP-43 SUMOylation: NaAsO2 (250 μM), D-Sorbitol (400 mM), NaCl (300 mM), Heat Shock (42 °C) in HEK293T (Immunofluorescence) and HEK293T HA-SUMO2 stable cells (biochemistry). One-Way ANOVA with Fisher’s LSD test. Data presented as mean ± SEM. *p<0.05, **p<0.005, ****p<0.0001. **(B)** Representative western blot from SUMOylation assays testing the selectivity of SUMO paralogs to SUMOylate TDP-43 in response to 1 hour sodium arsenite (250 μM) in HEK293T cells with transient expression of SUMO paralogs. **(C)** Representative GFP-Trap SUMOylation assay depicting increased SUMOylation with ALS-causing Q331K mutation in response to 250 µM sodium arsenite stress in HEK293T cells with transient expression of HA-SUMO2. Unpaired T-test. Data presented as mean ± SEM relative to stressed TDP-43-GFP (WT) condition, *p<0.05. **(D)** Representative images of SUMO2/3 and TDP-43 in the nucleus of mouse cortical neurons (7 DIV) in unstressed and 1 hour sodium arsenite (250 μM) stressed conditions. **(E)** Representative control imaged from Proximity Ligation Assay (PLA) in mouse primary cortical neurons. Scale bar = 20 μm. (N = 4). **(F)** Representative western blot and quantification from GFP-Trap SUMOylation assays testing the kinetics of TDP-43 SUMOylation in response to sodium arsenite stress (250 μM) and its relationship to insoluble phosphorylated TDP-43 in HEK293T HA-SUMO2 stable cells. (N = 3). Data presented as mean ± SEM relative to 3-hour stress condition. Linear regression analysis of Relative SUMOylation against time in response to stress. **(G)** Representative dose-response assay to detect TDP-43 SUMOylation after 1 hour of sodium arsenite stress (1 = 250 μM, 1/2 = 125 μM, 1/4 = 62.5 μM, etc.) in HEK293T HA-SUMO2 stable cells. One-Way ANOVA with Fisher’s LSD test. Data presented as mean ± SEM relative to 250 μM sodium arsenite (1) condition, *p<0.05, **p<0.005, ****p<0.0001.

**Fig. S2: Analysis of TDP-43 lysine to arginine mutation at K136 and K408. (A)** Representative fluorescent microscopy images expressing TDP-43-GFP with K136R and RNA binding deficient (5FL) mutations in HEK293T cells. Scale bar = 10 μm. **(B)** Predicted alignment error output from AlphaFold3 prediction of TDP-43 structure (residues 1-414) interacting with UG (x6) RNA (residues 415-426). **(C)** Predicted structure of TDP-43 with annotated domains. Color annotates predicted local difference test (plDDT)j scores. NTD = N-Terminal Domain; RRM1/RRM2 = RNA Recognition Motif 1/2; CTD = C-Terminal Domain. **(D)** Predicted structure of TDP-43^K408R^ highlighting no addition of secondary structure at the C-terminus surrounding residue 408 upon mutation. **(E)** Representative image and quantification of TDP-43-GFP expression in HEK293T cells denoting mislocalization in a subset of cells overexpressing TDP-43 with K408R mutation. (N = 3) Student’s T-test. Data presented as mean ± SEM.

**Fig. S3: Characterization of SUMO2 fusion to the C-terminus of TDP-43.** **(A)** Representative western blot and quantification of TDP-43-HA and TDP-43-SUMO2-HA expression in HEK293T cells. (N = 3) 2-way ANOVA with Fishers LSD test. Data presented as mean ± SEM relative to unstressed TDP-43-HA condition. *p<0.05, **p<0.005. **(B)** Representative immunofluorescence microscopy images and quantifications of TDP-43-HA and TDP-43-SUMO2-HA fusion in HEK293T cells in response to cellular stress (250 μM sodium arsenite for 1 hour). (N = 3) Student’s T-test. Data presented as mean ± SEM relative to unstressed TDP-43-HA condition, *p<0.05. Stress granule size is presented as nested data where each data point is a stress granule across 12-15 cells per biological replicate and the median stress granule size is presented with 95% CI error bars. T-test for nested data was performed (N = 3), **p<0.01.

**Fig. S4: Generation of the TDP-43^K408R^ mouse line. (A)** Schematic of the knock in approach to edit the endogenous *Tardbp* to express TDP-43^K408R^. **(B)** Schematic of LNA probe based genotyping approach and allelic discrimination assay to genotype TDP-43^K408R^ mouse line. **(C)** Sex and mendelian ratios of F3 generation TDP-43^K408R^ mice from K408R/+ and K408R/+ breeding pairs used to generate behaviour and histology cohorts. Observed vs Expected statistical analysis assuming an expected 1:1 ratio sex ratio and 1:2:1 mendelian ratio; no significant differences observed.

**Fig. S5: Characterizing the stress response in TDP-43^K408R^ primary cortical neuron cultures. (A)** Classification and quantification of G3BP1 stress granule (SG) intensity in response to 1 hour sodium arsenite stress (250 μM) and recovery. Data presented as mean ± SEM. (N = 5 per genotype) 2-Way ANOVA with Tukey’s multiple comparisons analysis. *p<0.05, ***p<0.0005. **(B)** Quantification of the localization of G3BP1 stress granules (SGs) in response to 1 hour sodium arsenite stress (250 μM) and recovery. Data presented as mean ± SEM. (N = 5 per genotype) 2-Way ANOVA with Tukey’s multiple comparisons analysis. ***p<0.0005. **(C)** Quantification of the percentage of neurons with positive TDP-43 nuclear foci in G3BP1 stress granule positive and G3BP1 stress granule negative neurons in response to 1 hour sodium arsenite stress (250 μM) and recovery. Data presented as mean ± SEM. (N = 5 per genotype) 2-Way ANOVA with Tukey’s multiple comparisons analysis. ***p<0.0005. **(D)** Quantification of the average number of TDP-43 nuclear foci in neurons positive for TDP-43 nuclear foci in response to 1 hour sodium arsenite stress (250 μM) and recovery. Data presented as mean ± SEM. (N = 5 per genotype) 2-Way ANOVA with Tukey’s multiple comparisons analysis. *p<0.05. For all analyses wild type neurons are presented in grey and K408R/K408R neurons are presented in purple.

**Fig. S6: Characterizing the effect of blocking TDP-43 SUMOylation in primary cortical neurons. (A)** Quantification of RIPA and UREA fractions from mouse primary cortical neurons (7 DIV) during stress (1 hour 250 μM sodium arsenite) and recovery related to Fig. 3B. N = 3-5, 2-Way ANOVA with Fisher’s LSD test. Data presented as mean ± SEM, * p<0.05, ** p<0.005. **(B-D)** Representative immunofluorescent images of primary cortical neurons (7 DIV) treated with 250 μM sodium arsenite for 1 hour then recovered for 3 hours to induce TDP-43 foci formation to analyze colocalization with nuclear markers: **(B)** HSPA1L was visualized through lentiviral transduction of HSPA1L-mRuby2; **(C)** paraspeckle marker PSPC-1; and **(D)** Ubiquitin. (N = 3). At least 10 cells positive for TDP-43 foci and foci of interest were imaged and analyzed per replicate. **(E)** Quantification of primary cortical neurons with TDP-43 mislocalization in response to repeated stress (30 minutes 250 μM sodium arsenite) and recovery (30 minutes washout) related to Fig. 3E. (N = 3) 2-Way ANOVA with Fishers LSD test. Data presented as mean ± SEM. **(F)** Representative western blot and analysis of RIPA soluble and UREA fractions from primary cortical neurons in response to chronic stress (30 hours of 15 μM sodium arsenite) and recovery. Data presented as mean ± SEM relative to WT for each condition. 2-Way ANOVA with Fishers LSD test. *p<0.05, **p<0.005. For all analyses wild type neurons are presented in Grey and K408R/K408R neurons are presented in purple.

**Fig S7: General wellness of TDP-43^K408R^ mice during development and aging. (A)** Genotype and sex composition of mice subject to behaviour testing. **(B)** Weight of mice at P21. Unpaired T-test (Males); Mann-Whitney test (Females and All) **(C)** Quantification of the duration hanging on a wire at P21. Mann-Whitney test (Males, Females, and All). **(D)** Survival curve for female TDP-43^K408R^ mice (Males found in Fig. 5d). Curve comparisons analyzed using Log-Rank test and Gehan-Breslow-Wilcoxon test. **(E)** Weights of male and female TDP-43^K408R^ mice from 3 weeks to 64 weeks of age. Mixed-effects analysis. **(F)** Hindlimb clasping scores of male and female TDP-43^K408R^ mice from 3 weeks to 64 weeks of age. Mixed-effects analysis. See Table S2 for raw data details on statistical tests. Data presented as mean ± SEM. For all analyses wild type mice are presented in grey and K408R/K408R mice are presented in purple.

**Fig. S8: Characterizing the cognitive and social behaviour of TDP-43^K408R^ mice.** Quantification of cognitive and social behaviors in the **(A)** Light/Dark Box, **(B)** Y-maze, **(C)** 3-Chamber Social Interaction, **(D)** Marble Burying, and **(E)** Tail Suspension behaviour tasks at 2, 9, and 16 months of age (2MO, 9MO, and 16MO, respectively) in male and female TDP-43^K408R^ mice. All behaviour tests were analyzed with a Mixed-Effects Model with Tukey’s multiple comparison analysis, see Table S2. Data presented as mean ± SEM. For all analyses wild type mice are presented in grey and K408R/K408R mice are presented in purple.

**Fig. S9: Characterizing the motor behaviour of TDP-43^K408R^ mice.** Quantification of motor abilities in the **(A)** Hanging Wire, **(B)** Grip Strength, and **(C)** Rotarod behaviour tasks at 2, 9, and 16 months of age (2MO, 9MO, and 16MO, respectively) in male and female TDP-43^K408R^ mice. **(D)** Digigait behaviour tasks at 16 months of age in male and female TDP-43^K408R^ mice. Behaviour tests were analyzed with 2-Way ANOVA or Mixed-Effects Models, see Table S2. Data presented as mean ± SEM. For all analyses wild type mice are presented in grey and K408R/K408R mice are presented in purple.

**Fig. S10: Characterizing the activity of TDP-43^K408R^ mice.** Quantification of **(A)** total distance traveled (cm) and time in center (s) during the Open Field behaviour test and **(B)** the relative ambulatory activity (males) of TDP-43^K408R^ mice at 2, 9, and 16 months of age (2MO, 9MO, and 16MO, respectively). Behaviour tests were analyzed with 2-Way ANOVA or Mixed-Effects models, see Table S2. Data presented as mean ± SEM. For all analyses wild type mice are presented in grey and K408R/K408R mice are presented in purple.

**Fig. S11: Absence of overt cortical neurodegeneration or neuroinflammatory phenotypes in TDP‑43^K408R/K408R^ mice.** Cresyl violet staining used to measure cortical thickness in the prefrontal cortex (PFC) and motor area 1 (M1) of female **(A)** and male **(B)** TDP-43^K408R/K408R^ mice and littermate controls. Cux1 and Ctip2 staining and quantification in the motor cortex of female **(C)** and male **(D)** TDP-43^K408R/K408R^ mice and littermate controls. Iba1 staining of microglia **(E, F)** and GFAP staining of astrocytes **(G, H)** in female **(E,G)** and male **(F,H)** TDP‑43^K408R/K408R^ mice and littermate controls. Quantification represents evaluation of N = 4 mice per genotype, per sex, per time point. Micrographs are representative of the 16-month time point. Student’s T-test. Data presented as mean ± SEM. ns denotes p>0.05. For all analyses wild type mice are presented in grey and K408R/K408R mice are presented in purple.

**Fig. S12: Characterizing the molecular and histological effects of blocking TDP-43 SUMOylation in vivo. (A)** Representative western blot and quantification of TDP-43 and phosphorylated TDP-43 in the RIPA and UREA fractions of the cortex from male mice at 2, 9, and 16 months of age (2MO, 9MO, and 16MO, respectively). Data presented as mean ± SEM. (N = 4 per genotype) 2-Way ANOVA with Fisher’s LSD test. **(B)** Representative western blot and quantification of TDP-43 and phosphorylated TDP-43 in the RIPA and UREA fractions of the cortex from male mice at 2, 9, and 16 months of age (2MO, 9MO, and 16MO, respectively). Data presented as mean ± SEM. (N = 4 per genotype) 2-Way ANOVA with Fisher’s LSD test. *p<0.05, **p<0.005, ***p<0.0005. **(C)** Quantitative RT-PCR from 16MO mouse cortex for splicing isoforms of *Mapt*. (N=8, 4 per sex, per genotype) 2-Way ANOVA with Fisher’s LSD Test. Data presented as mean ratios of ∆Ct normalized to wild type ± SEM. **p<0.005. **(D)** Quantification of TDP-43 mislocalization in the lumbar spinal cord of 9-month-old from Fig. 5a split by sex. Each datapoint represents the average of 4 serial sections 40 μm apart from an individual mouse. Unpaired t-test. * = p<0.05, ** = p<0.005. **(E)** Representative western blot and quantification of TDP-43 and phosphorylated TDP-43 in the RIPA and UREA fractions of the lumbar spinal cord at 2, 9, and 16 months of age (2MO, 9MO, and 16MO, respectively). Data presented as mean ± SEM. (N = 8 per genotype; 4 per sex/genotype) 2-Way ANOVA with Fisher’s LSD test, *p<0.05, ***p<0.0005, ****p<0.0001 **(F)** Example images of neuromuscular junctions (NMJs) for scoring criteria to assess fully innervated, partially innervated, and fully denervated NMJs. **(G)** Representative images at 16 months of age (16MO) and quantification (2MO, 9MO, and 16MO) of neuromuscular junction (NMJ) innervation in the tibialis anterior of female TDP-43^K408R^ mice. >80 NMJs were quantified per animal. Data presented as mean ± SEM. (N = 3-4 per genotype) 2-Way ANOVA with Tukey’s multiple comparison analysis. **(H)** Representative images and quantification of ChAT+ motor neurons in the ventral horn of the lumbar spinal cord of male and female mice. Each datapoint is the average of 4 serial sections spaced 40 μm apart through the lumbar enlargement of the lumbar spinal cord. Data presented as mean ± SEM. Unpaired t-test, ** p<0.005. For all analyses wild type mice are presented in grey and K408R/K408R mice are presented in purple.

**Fig. S13: Optimization of proximity ligation assay in human tissue. (A)** Quantification of neuropathology score of PPFE prefrontal cortex (PFC) tissue sections (5um) from patients diagnosed with ALS/FTD and unaffected controls. Data presented as mean ± SEM. (N = 6-8) Unpaired t-test, ** p<0.005. **(B)** Representative images of proximity ligation assay (PLA) between TDP-43 and SUMO2/3 in human PPFE hippocampus tissue sections (5 µm). Yellow arrows indicate TDP-43:SUMO2/3 interactions where the signal specific to the PLA channel and not identified in the autofluorescence channel. Scale bar = 18 μm.
